# About miRNAs, miRNA seeds, target genes and target pathways

**DOI:** 10.18632/oncotarget.22363

**Published:** 2017-11-09

**Authors:** Tim Kehl, Christina Backes, Fabian Kern, Tobias Fehlmann, Nicole Ludwig, Eckart Meese, Hans-Peter Lenhof, Andreas Keller

**Affiliations:** ^1^ Center for Bioinformatics, Saarland University, Saarland Informatics Campus, Saarbrücken, Germany; ^2^ Chair for Clinical Bioinformatics, Saarland University, Saarland Informatics Campus, Saarbrücken, Germany; ^3^ Department of Human Genetics, Saarland University, Homburg, Germany

**Keywords:** non-coding RNA, systems biology, target gene, microRNA

## Abstract

miRNAs are typically repressing gene expression by binding to the 3’ UTR, leading to degradation of the mRNA. This process is dominated by the eight-base seed region of the miRNA. Further, miRNAs are known not only to target genes but also to target significant parts of pathways. A logical line of thoughts is: miRNAs with similar (seed) sequence target similar sets of genes and thus similar sets of pathways.

By calculating similarity scores for all 3.25 million pairs of 2,550 human miRNAs, we found that this pattern frequently holds, while we also observed exceptions. Respective results were obtained for both, predicted target genes as well as experimentally validated targets. We note that miRNAs target gene set similarity follows a bimodal distribution, pointing at a set of 282 miRNAs that seems to target genes with very high specificity. Further, we discuss miRNAs with different (seed) sequences that nonetheless regulate similar gene sets or pathways. Most intriguingly, we found miRNA pairs that regulate different gene sets but similar pathways such as miR-6886-5p and miR-3529-5p. These are jointly targeting different parts of the MAPK signaling cascade.

The main goal of this study is to provide a general overview on the results, to highlight a selection of relevant results on miRNAs, miRNA seeds, target genes and target pathways and to raise awareness for artifacts in respective comparisons. The full set of information that allows to infer detailed results on each miRNA has been included in miRPathDB, the miRNA target pathway database (https://mpd.bioinf.uni-sb.de).

## INTRODUCTION

miRNAs are small non-coding RNAs [1] that are well conserved between different organisms [2, 3]. Since the discovery of the first miRNAs, over 2,500 human and a total of 28,645 miRNAs have been stored in the miRBase [4, 5], which is now in its 21^st^ version (last update in July 2014). Recently, we published the miRCarta repository (https://mircarta.cs.uni-saarland.de/), hosting information on 43,699 miRNAs and miRNA candidates, 364,647 target genes with experimental evidence and 11 million predicted target genes. Typically, miRNAs down-regulate genes, so called target genes, by binding to the 3’ UTR of the respective targets [6, 7]. The binding has however not to be perfect across the whole mature miRNA sequence: in mammals it is dominated by the so-called seed region [8]. This seed region at the 5’ end of the mature miRNA consists of eight nucleotides. Various other factors have been found to add to a strong seed binding. These include additional base pairs towards the 3’ end of the mature miRNA sequence [9]. Also factors such as the total number of binding sites of a miRNA at the 3’ UTR, the proximity to the gene start or the local AU composition are known to impact the targeting of a gene [9]. These features have been incorporated in computational approaches to predict targets of miRNAs [10], including miRanda [11], mirSVR [12], DIANAmicroT [13], targetscan [14] and others.

Given approximately 2,500 human mature miRNAs and 22,500 human protein coding genes, 50 million potential pair-wise interactions between miRNAs and genes are possible. Information on these, partially predicted by using the computational tools mentioned above, but also experimentally validated miRNA-gene target pairs are stored in target databases such as miRTarBase [15]. Especially the miRNA-target interactions that have been validated by accurate low-throughput approaches such as luciferase reporter assays are enriched for miRNA-target pairs with well-studied miRNAs and genes, predominantly known from oncological research. As a consequence, an unbiased comparison of these relationships is difficult. Nonetheless we performed our calculations for both, predicted and experimentally validated target genes. Beyond the targeting of single genes, miRNAs are known to specifically regulate pathways [16]. Information on 2,571 human miRNAs targeting 2,565 biochemical categories (totaling around 20 Million interactions) has been stored in the miRPathDB (https://mpd.bioinf.uni-sb.de) [17]. This database does not only list target genes for each miRNA, but has been implemented as central repository for storing information on target pathways and related data on all human miRNAs.

When considering the miRNA biogenesis and how miRNAs regulate genes, a logical line of thoughts is that miRNAs with similar overall sequence also have similar seed sequences. Thus, they are expected to regulate similar sets of target genes and consequently the same set of target pathways. In this study, we investigated this hypothesis by analyzing the similarity of miRNA sequence, seed sequence, target gene sets and target pathways for all 3.25 million pairs of 2,550 miRNAs. We were especially interested in cases where the expected pattern is not observed, this includes among others: 1) miRNAs with high overall similarity, but rather low seed similarity and the opposite behavior. 2) miRNAs with low seed similarity, but similar target gene sets and target pathways and the opposite behavior and 3) miRNAs with different target gene sets, but nonetheless similar target pathways as well as the opposite behavior. Because of the bias in experimentally validated target genes mentioned above, we focus on the union of predicted miRNA target interactions extracted from miRPathDB. Nonetheless, selected results for experimentally validated miRNA target genes and target pathways have also been calculated and compared to the results obtained for predicted target genes and the respective pathways.

## RESULTS

### Distribution of miRNA, seed, target gene and target pathway similarity

**Figure 1:**
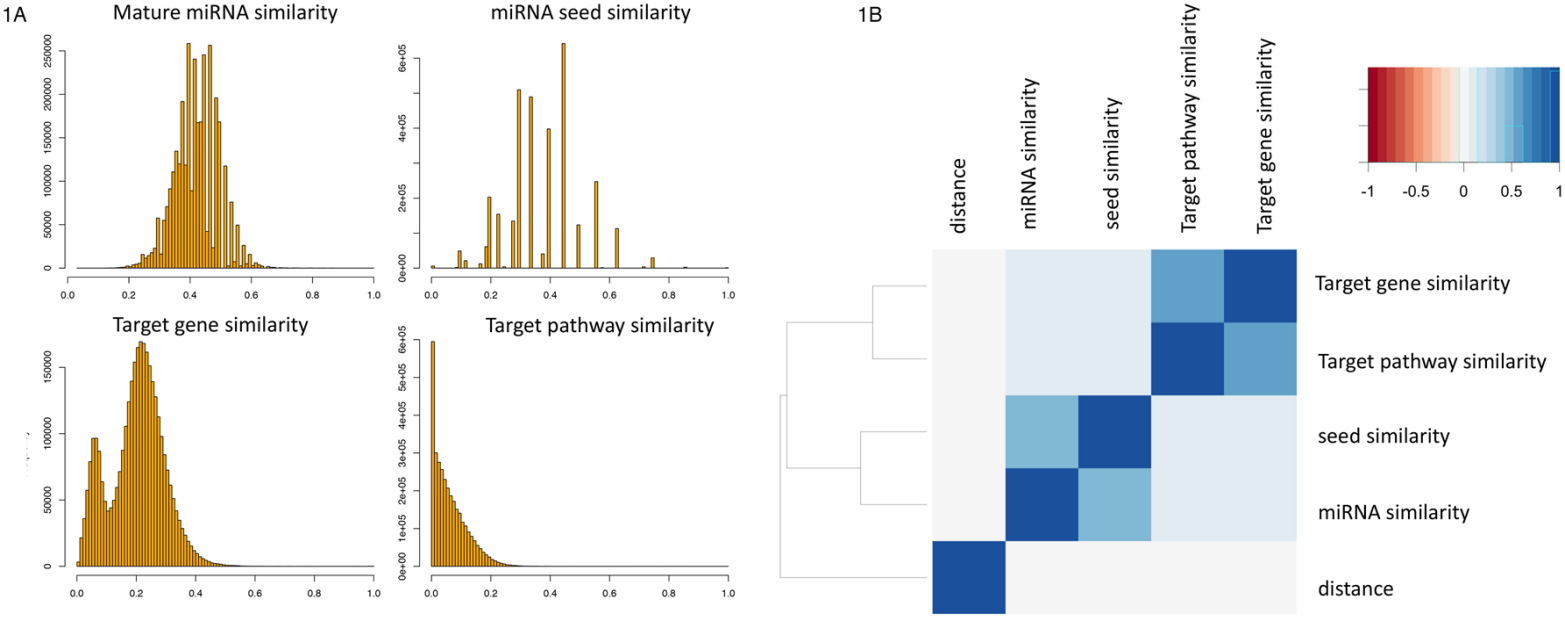
**(A)** For all 3. 25 Million pairs of miRNAs the similarity of the sequence, the seed sequence, target gene sets and target pathways is presented as histograms. Similarity scores (described in the Methods section) are in the range between 0 (no similarity) and 1 (identical). The bin width has been set to 0.01 (maximum of 100 bars per histogram). **(B)** Clustered correlation matrix. The correlation of the considered features are shown as heat map with dendrogram on the left side. Highest correlation was obtained for target pathway similarity compared to target gene set similarity followed by miRNA similarity to miRNA seed similarity.

As detailed in the Methods section, we calculated for all 3.25 million pairs of 2,550 miRNAs the similarity of the mature sequences, the seed sequences and the overlap in target genes as well as target pathways. Before comparing the results for the different similarity measures, we first investigated the distribution of the features. For all four measures, the distributions of the 3.25 million values for all miRNA pairs are presented in Figure 1A. While the distribution of the miRNA similarity and miRNA seed similarity approximated a normal distribution, we observed especially for the miRNA target gene sets a bimodal distribution. The left part of the distribution contains miRNAs that have overall low overlap in target gene sets to other miRNAs. These represent miRNAs that target genes in a rather specific manner. The right part of the distribution contains miRNAs with high similarity in target gene sets to many other miRNAs, pointing at lower specificity in the targeting process. By using a median Jaccard index (JI) of 0.1 as threshold, we observed 282 miRNAs that are specific regulators. On the opposite end, the remaining 2,316 miRNAs had higher median JI, indicating that their target gene sets were less specific. The miRNA with overall highest median JI to all other miRNAs was hsa-miR-4668-5p (median JI of 0.3). While we did not observe a bias with respect to the discovery date for miRNAs in both groups, the decreased specificity for the 2,316 miRNAs can however be explained partially by larger sets of target genes for respective miRNAs.

A cluster heat map of all above features plus the genomic distance between the miRNA pairs is presented in Figure 1B. It shows that a high correlation between seed and miRNA similarity (Pearson correlation of 0.51) has been observed as well as a high similarity between target gene sets and target pathways (Pearson correlation of 0.6). On an overall scale, no correlation of these features with the genomic distance is found. Both, for the miRNA sequence and the seed sequence similarity, a slightly increased correlation to target pathways is computed (Pearson correlation of 0.08 and 0.1) compared to the target genes (Pearson correlation of 0.07 for both variables). Still, these correlation values were highly significant, also driven however by the large number of observations (p < 10^-10^).

### Seed and miRNA similarity

**Figure 2:**
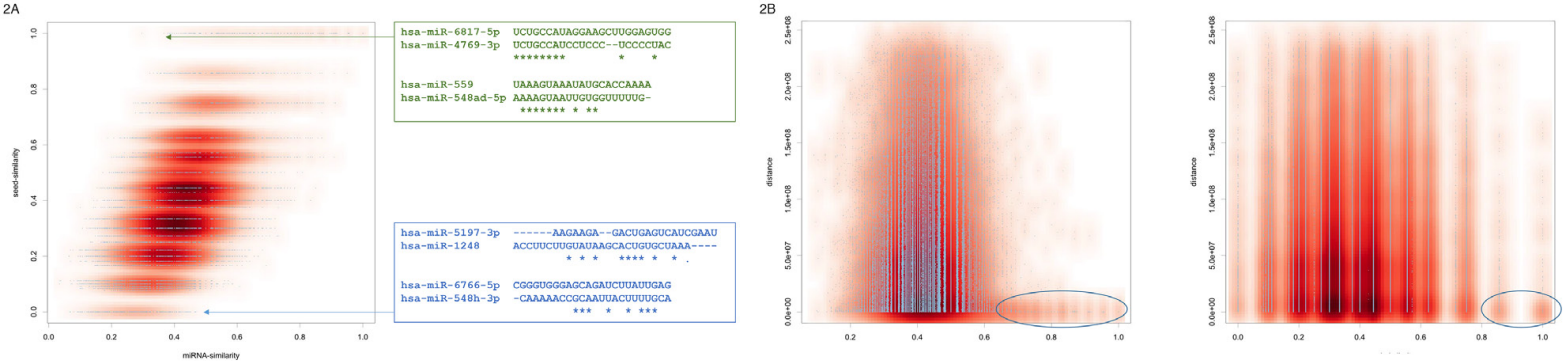
**(A)** Scatter plot of miRNA sequence similarity score compared to miRNA seed similarity score. Since the plot contains 3.25 million data points, the density distribution is also shown in red. The green and blue sequences represent extreme examples, i.e. miRNAs with high sequence similarity compared to the seed similarity (blue) and miRNAs with high seed sequence similarity compared to the overall lower sequence similarity (green). **(B)** Scatterplots for the sequence similarity and the seed similarity, each compared to the genomic distance. The blue ellipses highlight miRNAs with similar sequences and similar seeds, respectively, that also have close genomic proximity.

Since the eight-base seed region of the miRNA determines around one third of the total average length of 21-23 nucleotides it is evidential that similar seed regions have to correlate to a certain amount with similar miRNA sequences. As Figure 2 shows this expected correlation is indeed observed: the Pearson correlation coefficient for both variables is 0.51. While it is obvious that miRNAs with almost the same sequence usually also have similar seed sequences (data points in the upper right corner) and miRNAs with totally different sequences have mostly also different seeds (data points in the lower left corner), we specifically investigated extreme cases: miRNAs with fairly dissimilar mature sequences but still high seed similarity as well as miRNAs with overall quite high mature sequence similarity but different seed sequences. Two extreme examples for both cases are included in Figure 2A. The green sequences represent miRNAs with overall dissimilar sequences but similar seed while the blue miRNAs exhibit the opposite case. For all pairs of miRNAs located on the same chromosome we asked whether either seed or miRNAs similarity are correlated to the genomic distance. As Figure 2B details for both features no clear correlation with the distance between miRNAs can be observed. Nevertheless, especially for miRNAs with overall very high similarity a tendency for the expected genomic clustering is observed (highlighted in Figure 2B).

### Seed similarity and target gene / pathway similarity

**Figure 3:**
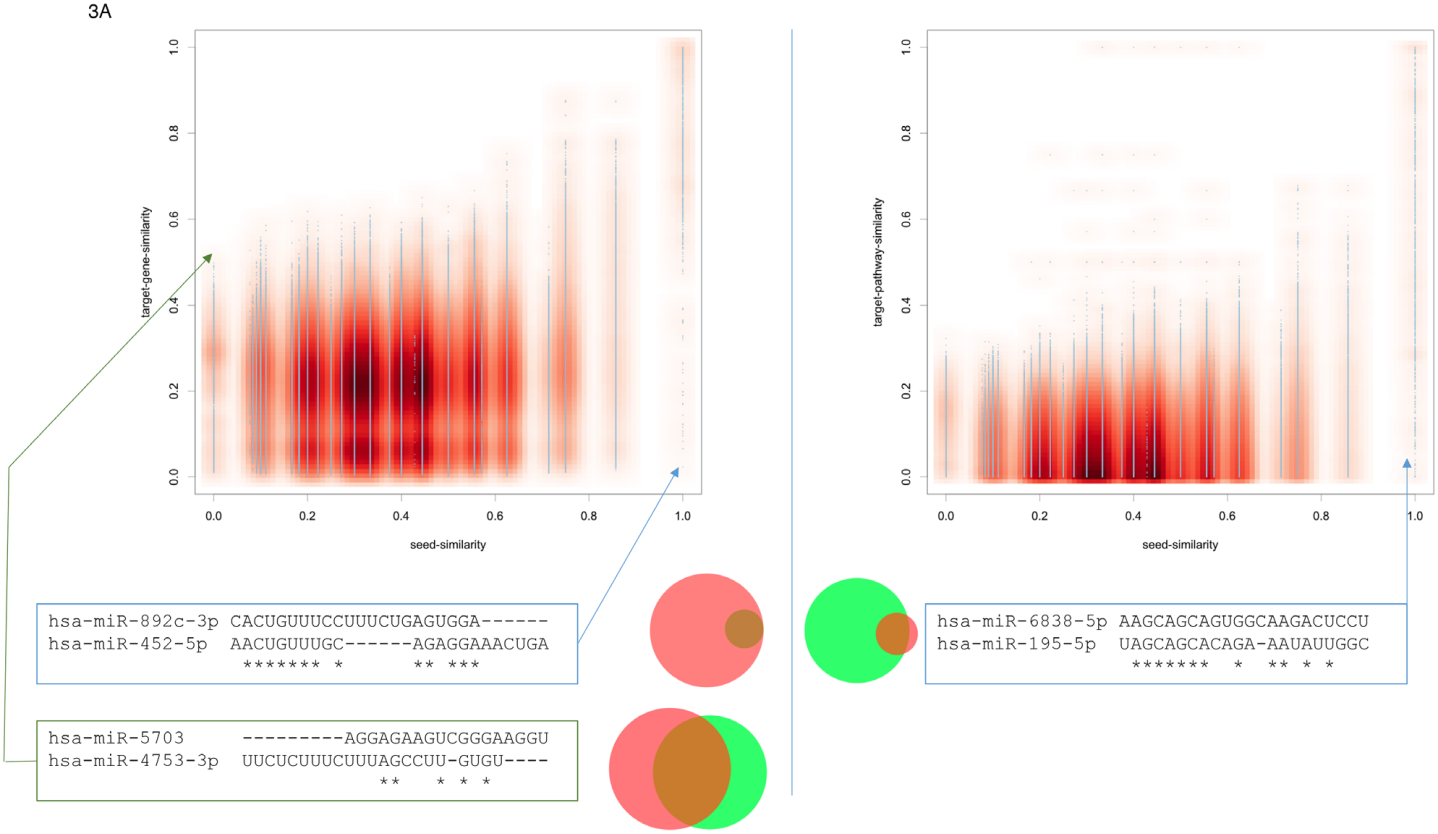
**(A)** Scatter plots for the seed similarity compared to the target gene set similarity (left part) and the target pathway set similarity (right part). Again, extreme cases not following the expected pattern are highlighted. The area proportional Venn diagrams highlight the overlap in the gene set and the pathway set for the respective examples.

Next, we compared the influence of the seed similarity to the target gene and the target pathway similarity for all pairs of miRNAs. As mentioned in the first paragraph of the results section the seed sequence correlated with both, gene set and pathway similarity. The correlation with the target pathway similarity was even slightly higher as compared to the target gene set similarity. Scatter plots for both cases are presented in Figure 3A. Again, while the information on all 3.25 Million pairs is available in miRPathDB we here pick extreme examples.

Similar to the previous consideration with respect to the similarity of mature miRNAs and their seeds, we also investigated the extreme cases not following the expected pattern, i.e. high seed similarity leads to high target gene set similarity. Given completely dissimilar seeds, the pair hsa-miR-5703 and hsa-miR-4753-3p for example yields a significant overlap of target genes (Jaccard index (JI) of 0.4; green box in the lower left part of Figure 3A). In this and other such extreme cases we observed a bias towards larger miRNA target gene sets. In the concrete case, miR-4753-3p is predicted to target 9,267 genes and miR-5703 to target 8,100 genes. The overlap between both sets is 4,975 genes. The opposite example is the pair hsa-miR-892c-3p and hsa-miR-452-5p. The seeds of both miRNAs have a very high similarity score, but the JI between the target gene sets is only 0.12. In this case, the low JI is explained by the fact that the target gene set of the second miRNA is a very small subset of the target gene set of the first miRNA. Generally, we observed a bias for miRNA pairs where one miRNA has a large and the second miRNA a comparably small target gene set.

Comparing miRNA seed similarity to miRNA target pathway similarity (right part of Figure 3A), we observe that no miRNA pair with low seed similarity has a high target pathway similarity. Vice versa, miRNAs with high seed similarity partially had different miRNA target pathway sets. One example is presented below the corresponding scatter plot in Figure 3A: hsa-miR-6838-5p and hsa-miR-195-5p have high seed similarity scores, still very similar overall sequences but the target pathways of the second miRNA are almost a subset of the targets of the first miRNA. Similar patterns were also observed for target genes (left part of Figure 3A). The results describing the similarity of mature miRNA sequences to target gene sets and target pathway sets were largely consistent with those for the miRNA seeds just described. They are included in the online version of miRPathDB and presented as scatter plots in Supplementary Figure 1.

### Target gene sets and target pathways

**Figure 4:**
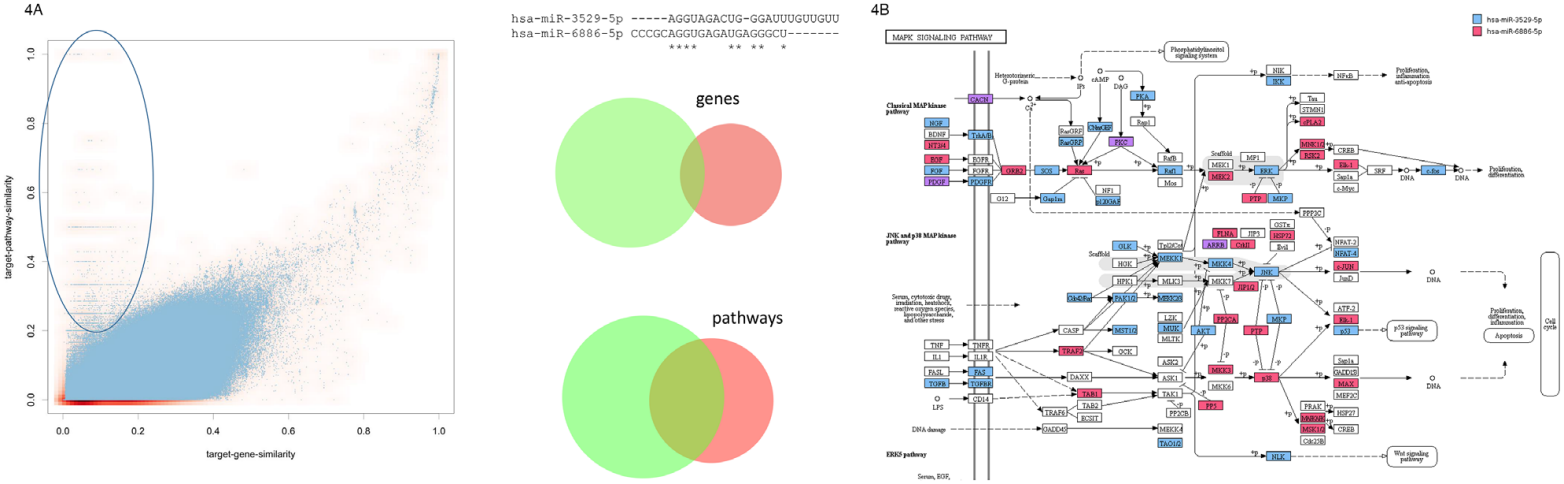
**(A)** Scatter plot for the target gene set and the target pathway similarity. The ellipse in the upper left part highlights miRNA pairs with overall low target gene similarity and high target pathway set similarity. The example on the right side presents two miRNAs (hsa-miR-3529-5p and hsa-miR-6886-5p) with low target gene similarity and significant target pathway similarity, as demonstrated by the area proportional Venn diagrams. **(B)** For the two miRNAs from Figure 4A the targeting of the KEGG MAPK signaling cascade is detailed. Targets of miR-3529-5p are highlighted in blue, targets of miR-6886-5p in red and joint targets of both miRNAs in purple. As the figure shows, both miRNAs target specific parts of the pathway such that overall a very large fraction of the path is targeted while the overlap between both miRNAs is small.

Finally, we asked on the similarity of miRNA target gene sets and miRNA target pathways. The corresponding scatter plot on the 3.25 million pairs is presented in Figure 4A. In addition, we computed the overall strongest correlation among all possible comparisons for these two features (Pearson correlation of 0.77, see also Figure 1B). Detailed inspection of the scatter plot highlights that, as expected, no miRNA pairs with similar target gene sets but different target pathway sets existed. Vice versa, we found a substantial number of miRNA pairs with divergent target gene sets but targeting the same pathways (highlighted in Figure 4A). The right panel of Figure 4A presents one such example: miR-6886-5p and miR-3529-5p. Both have low seed and miRNA sequence similarity scores and, also as expected, target different gene sets. Nonetheless we observed a substantial overlap in target pathway sets. The targeted KEGG pathways and chromosomes are shown in Supplementary Figure 2. While a substantial overlap of the targeted KEGG pathways has been detected, different chromosomes are enriched with target genes of the two miRNAs. A detailed inspection of the targeted pathways highlights that the miRNAs jointly regulate different parts of the signaling cascades. Exemplarily, the MAPK signaling cascade is presented on the right panel of Figure 4B. While the MAPK signaling cascade is targeted nearly completely by these two miRNAs, only 6 target genes overlap between them.

We repeated the same calculations for experimentally validated miRNA target gene pairs. Here, we took both, targets with weak experimental evidence (e.g. microarrays) and strong experimental evidence (e.g. reporter assays) from miRTarBase into account. We also calculated the results for the joint set of experimental targets with strong and weak evidence. The three scatter plots are presented in Supplementary Figure 3. In these comparisons we again observed a substantial number of miRNA pairs with low target gene set similarity but high target pathway similarity, comparable to the predicted targets (highlighted in blue in Supplementary Figure 3). Especially for the strong evidence targets the positive correlation obtained for the predicted targets has been generally lost however. This may be due to the nature of the experiments: for one miRNA usually not the complete targetome is deciphered but only a subset of the most interesting targets. Respectively, the target gene sets per miRNA are typically less sensitive but more specific as compared to the predicted target gene sets. As an example of a miRNA pair with different targeted genes (9 genes and 4 genes of which one gene overlaps) however located on the same pathway we selected let-7f-5p and miR-138-5p, jointly targeting the p53 signaling cascade (Supplementary Figure 4).

### Most variable miRNA pairs

Our initial hypothesis was that miRNAs with similar seed and similar overall sequence have similar target gene sets and lead to similar target pathway sets. This means that either the four similarity scores of a miRNA pair should all be high or low. The interesting examples are those where the four similarity scores are differing, e.g. low miRNA and seed similarity but high target pathway similarity. We thus calculated the miRNA pairs having the overall highest heterogeneity in terms of their mature sequence similarity, seed sequence similarity, target gene set similarity and target pathway similarity by computing the variance of the four similarity scores. The 30 miRNA pairs with the highest variance are detailed in Table 1. All of these are characterized by a high seed sequence similarity, still an excellent overall mature sequence similarity, but already a decreased target gene set similarity and lowest target pathway similarity. For respective pairs, we observed one other key characteristics: a usually close genomic proximity. Indeed, this can already be well quantified by comparing the co-localization on the same chromosomes. While of the 3.25 million pairs we considered 0.167 million were on the same chromosome (5.1%) and 3.082 Million were on different chromosomes (94.9%), 12 of the 30 miRNAs shown in Table 1 are located on the same chromosome (40%). As the genomic distance in the fourth column reveals, most miRNA pairs were separated only by few thousand bases.

**Table 1:** 30 miRNA paris with highest overall variance

miRNA pair	mature similarity	seed similarity	chr. Distance	target gene similarity	target pathway similarity	shared pathways
**hsa-miR-526b-3p hsa-miR-518c-3p**	0,75	0,86	14332	0,08	0,018	5
**hsa-miR-526b-3p hsa-miR-518f-3p**	0,83	0,86	5603	0,07	0,004	1
**hsa-miR-485-5p hsa-miR-6884-5p**	0,68	1,00	NA	0,19	0,012	2
**hsa-miR-215-5p hsa-miR-192-5p**	0,86	1,00	NA	0,58	0,000	0
**hsa-miR-3681-3p hsa-miR-216a-3p**	0,56	1,00	NA	0,12	0,002	1
**hsa-miR-487a-3p hsa-miR-487b-3p**	0,86	0,86	5968	0,03	0,010	1
**hsa-miR-6088 hsa-miR-4770**	0,55	1,00	NA	0,10	0,000	0
**hsa-miR-6088 hsa-miR-143-3p**	0,54	1,00	NA	0,10	0,000	0
**hsa-miR-7153-5p hsa-miR-146a-5p**	0,63	1,00	NA	0,07	0,019	4
**hsa-miR-7153-5p hsa-miR-146b-5p**	0,63	1,00	NA	0,07	0,012	3
**hsa-miR-892c-3p hsa-miR-4676-3p**	0,56	1,00	NA	0,11	0,031	8
**hsa-miR-892c-3p hsa-miR-452-5p**	0,50	1,00	5966897	0,12	0,030	9
**hsa-miR-181a-3p hsa-miR-181a-2-3p**	0,83	0,75	NA	0,06	0,007	1
**hsa-miR-518e-3p hsa-miR-520a-3p**	0,71	0,86	38937	0,06	0,023	5
**hsa-miR-3064-5p hsa-miR-6504-5p**	0,71	1,00	NA	0,08	0,007	1
**hsa-miR-4782-3p hsa-miR-6766-3p**	0,54	1,00	NA	0,14	0,000	0
**hsa-miR-128-3p hsa-miR-216a-3p**	0,59	1,00	NA	0,12	0,003	1
**hsa-miR-106a-5p hsa-miR-17-5p**	0,92	1,00	NA	0,33	0,065	33
**hsa-miR-524-3p hsa-miR-518d-3p**	0,82	0,75	23855	0,05	0,017	1
**hsa-miR-519d-3p hsa-miR-518c-3p**	0,76	0,86	4582	0,08	0,015	7
**hsa-miR-519d-3p hsa-miR-518b**	0,79	0,86	10592	0,08	0,013	6
**hsa-miR-519d-3p hsa-miR-518f-3p**	0,71	0,86	13313	0,07	0,002	1
**hsa-miR-6807-3p hsa-miR-217**	0,57	1,00	NA	0,10	0,019	2
**hsa-miR-1273c hsa-miR-187-5p**	0,68	0,86	NA	0,05	0,000	0
**hsa-miR-147b hsa-miR-147a**	0,86	0,86	NA	0,11	0,050	15
**hsa-miR-10a-5p hsa-miR-100-5p**	0,80	0,75	NA	0,04	0,000	0
**hsa-miR-6788-3p hsa-miR-197-3p**	0,68	0,86	248956422	0,06	0,000	0
**hsa-miR-20a-5p hsa-miR-17-5p**	0,91	1,00	432	0,37	0,096	33
**hsa-miR-370-5p hsa-miR-1193**	0,50	1,00	118889	0,02	0,000	0

## DISCUSSION

In the present study, we investigated the influence of seed similarity, miRNA similarity, target gene set similarity and target pathway similarity. In our consideration, we included all 3.25 million pairs of human miRNAs as annotated in the current miRBase version V21. One of the major strength of our study is at the same time one of the major drawbacks: since our ambition was to avoid the severe bias by restricting to miRNA – target gene pairs with experimental validation that are enriched for few central miRNAs and also target genes, known mostly from oncological research, we focused on predicted target genes. This avoids the aforementioned challenge of using validated target genes but also introduces bias by target predictors. We relied in our calculations on the union of the predicted targets that have been extracted from the latest implementation of miRPathDB.

One of the most important aspects in this work was to raise awareness of potential artifacts. We thus critically asked which results may be due to bias. First, with respect to the bimodal distribution of pair-wise target gene set similarities we also found a correlation to the size of the target gene sets. Another example are miRNA pairs with high seed similarity but low target gene set or target pathway similarity. Such pairs are frequently observed if one miRNA has a broad target gene and / or target pathway set while the other miRNA has a smaller target gene or target pathway set, which is however almost completely a subset of the first miRNAs sets. Here, it is hard to distinguish without thorough experimental evidence whether corresponding effects are real or are due to the target prediction. Among the most interesting findings in our study were pairs of miRNAs with high seed and mature sequence similarity having however different target pathways. Such pairs were frequently closely co-localized in the genome. These results depend on the performance of target prediction programs. Usually, one would expect similar miRNAs to have nearly identical target gene sets and target pathways. One reason for considering genomic clusters of miRNAs targeting the same genes and pathways are evolutionary aspects [18].

Among the most important results of our analyses we also observed that miRNAs can generally have different target gene sets but at the same time regulate the same pathway. Here, usually different sub-networks of the same network are regulated. One of the most significant examples was the pair consisting of miR-6886-5p and miR-3529-5p. Both of them have different seed and miRNA sequences and, also as expected, target different gene sets but both target the MAPK signaling cascade.

While we focused on predicted target genes of miRNAs we also included results on experimentally validated targets (both, strong and weak evidence targets). A direct comparison between validated and predicted pathways is however constrained by the nature of the validation experiments. Especially if low throughput reporter assays for single miRNAs are applied, usually not the complete targetome is deciphered but only a subset of the most interesting targets. Respectively, the target gene sets per miRNA are expected to be less sensitive but more specific as compared to the predicted target gene sets. Nonetheless, we detected examples where validated target gene sets are different but the target pathways are similar. Here, it has to be kept in mind that only positive miRNA target gene relations are taken into account. For respective pairs where miRNAs target different gene sets and different parts of the same pathway, experiments that also demonstrate that genes on this pathway are actually targeted only by one of the miRNAs and not by the other would further support the hypothesis.

Of course, it is impossible to describe all interesting details analyses of 3.25 million pairs of miRNAs in this manuscript. We thus restrict ourselves to selected examples. The full set of information is available through the miRNA pathway dictionary web repository: https://mpd.bioinf.uni-sb.de/. Here, miRNAs with similar seed sequence, similar sequence, co-localized miRNAs, miRNAs with similar target gene sets or miRNAs with similar target pathway sets can be obtained with minimal effort. All data are available in CSV format but also as XLS files.

## MATERIALS AND METHODS

### Predicted miRNA targets

All miRNA target interactions (MTIs) were retrieved from predefined datasets provided by DIANA [13], miRDB [19] and TargetScan [14]. For consistency reasons, we mapped all miRNA identifier to miRBase [4, 20] version 21 and all target gene identifier to official gene symbols. We then combined the datasets of all three databases.

### Validated miRNA targets

In total we analyzed 2598 miRNAs that together have 14773 experimentally validated target genes that have been extracted from the miRTarBase. Among them 491 miRNAs with 2068 targets that are validated with strong experimental evidence and 2598 miRNAs with 14773 targets that are validated with weak experimental experiments.

### Similarity of mature miRNA and seed sequences

Mature miRNA sequences were retrieved from miRBase 21 [4, 20] and miRNA seed sequences were retrieved from TargetScan 7.1 [14]. The similarities between all pairwise sequences were calculated by aligning all sequence pairs using the edit-distance. For each alignment, we then computed the sequence similarity as fraction of matching bases and the length of the alignment.

### Defining miRNA target gene set similarity and target pathway similarity

Similarities of target gene and target pathway sets between all miRNA pairs were defined using the pairwise

$$\mathrm{Jaccard index}\;\mathrm{JI}\left(M,N\right)=\left|\frac{M\cap N}{M\cap N}\right|.$$

### Used resources for pathway enrichment analyses

All information about signaling pathways and functional categories in miRPathDB were extracted from the GeneTrail2 webserver [21], building on the GeneTrail framework [22]. In particular, we used the following databases: Biocarta, Chromosomes and Cytogenic bands, Gene Ontology [23], KEGG [24], NCI Pathway interaction database (PID) [25], Pfam [26], Reactome [27] and WikiPathways [28].

### Statistical analysis

To process all pairwise similarity measures for miRPathDB, we used the Python programming language (Version 2.7.6) and the biopython library (Version 1.69) [29]. For the statistical analysis, we used R programming environment (Version 3.0.2).

## SUPPLEMENTARY MATERIALS FIGURES


