# Supplementary material for: About miRNAs, miRNA seeds, target genes and target pathways
Source: Oncotarget. 2017 Nov 9;8(63):107167–75. doi: 10.18632/oncotarget.22363 (PMC5739805; doi:10.18632/oncotarget.22363)
Supplement: Supplementary file 1 [file oncotarget-08-107167-s001.pdf]

# About miRNAs, miRNA seeds, target genes and target pathways

## SUPPLEMENTARY MATERIALS

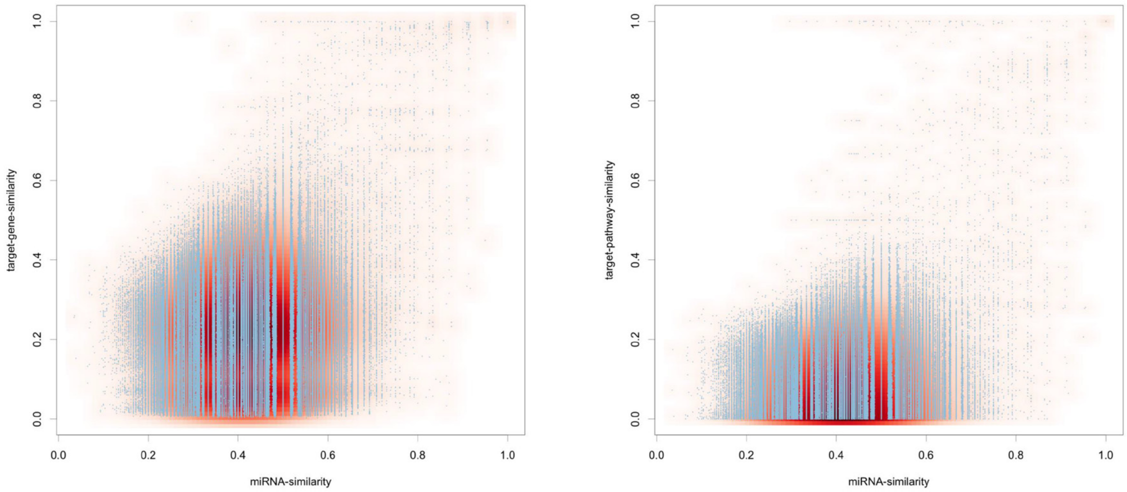


**Supplementary Figure 1: Scatter Plot fort he miRNA similarity versus target gene similarity (left) and miRNA similarity versus target pathway similarity (right).**

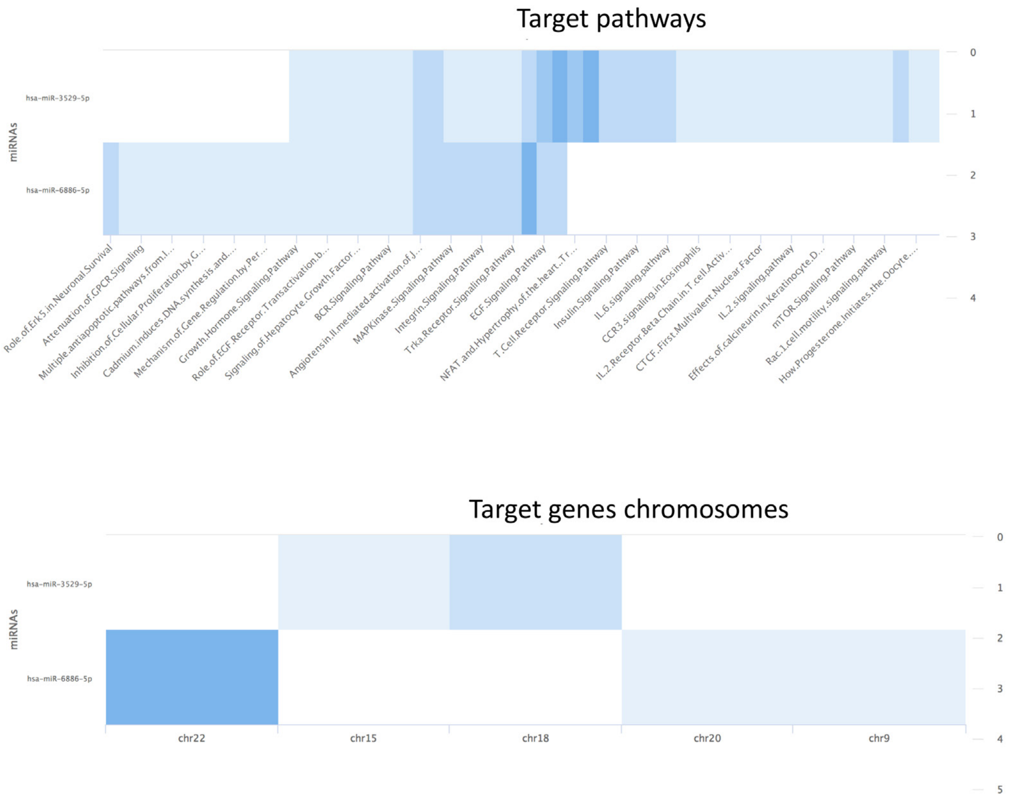


**Supplementary Figure 2: Heat map for two miRNAs and target pathways (upper part) and chromosomes (lower part).**

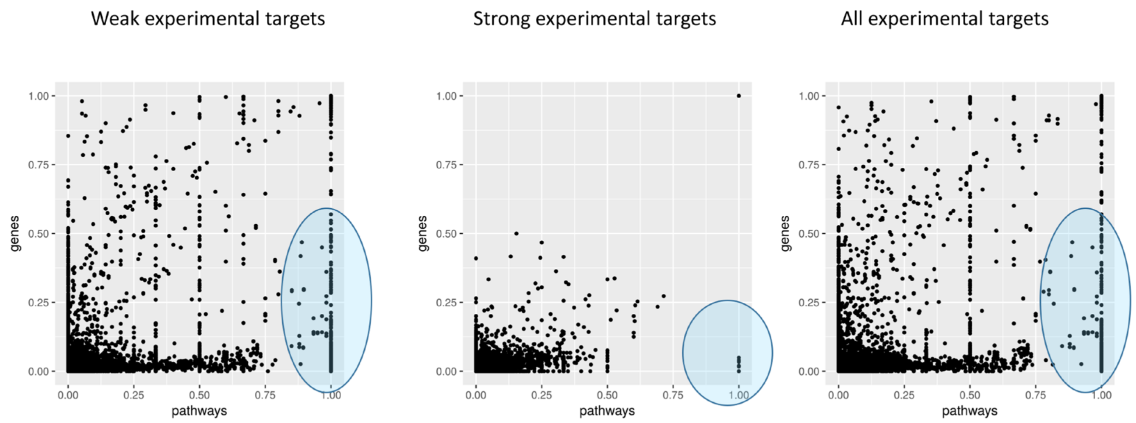


**Supplementary Figure 3: Scatter plots of target pathway versus target gene gene similarity for weak evidence targets (left panel), strong evidence targets (middle panel) and all targets with experimental evidence (right panel).** The area of interest described in the manuscript is highlighted in blue.

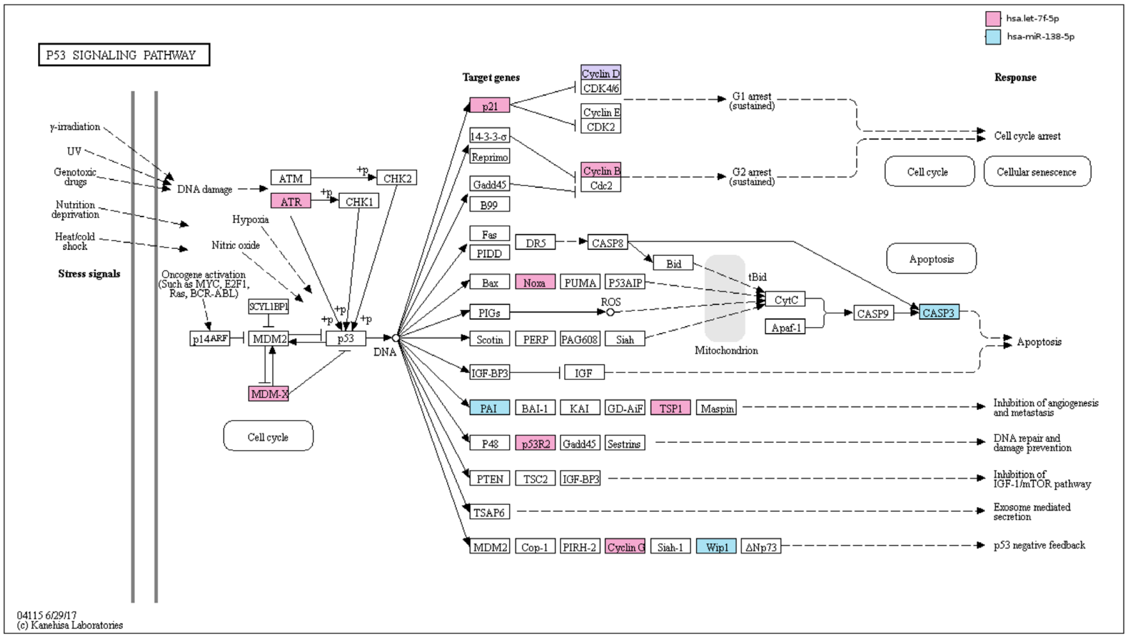


**Supplementary Figure 4: experimental targets of the two miRNAs on the p53 signalling cascade.** Targets are highlighted in different colors, only one purple target overlaps between both miRNAs.
